# Epidemiology of Meropenem/Vaborbactam Resistance in KPC-Producing *Klebsiella pneumoniae* Causing Bloodstream Infections in Northern Italy, 2018

**DOI:** 10.3390/antibiotics10050536

**Published:** 2021-05-06

**Authors:** Paolo Gaibani, Donatella Lombardo, Linda Bussini, Federica Bovo, Beatrice Munari, Maddalena Giannella, Michele Bartoletti, Pierluigi Viale, Tiziana Lazzarotto, Simone Ambretti

**Affiliations:** 1 Operative Unit of Clinical Microbiology, IRCCS Azienda Ospedaliero-Universitaria di Bologna, 40138 Bologna, Italy; donatella.lombardo@aosp.bo.it (D.L.); federica.bovo@aosp.bo.it (F.B.); beatrice.munari@gmail.com (B.M.); tiziana.lazzarotto@unibo.it (T.L.); simone.ambretti@aosp.bo.it (S.A.); 2 Operative Unit of Infectious Diseases, IRCCS Azienda Ospedaliero-Universitaria di Bologna, 40138 Bologna, Italy; linda.bussini@gmail.com (L.B.); maddalena.giannella@unibo.it (M.G.); m.bartoletti@unibo.it (M.B.); pierluigi.viale@unibo.it (P.V.)

**Keywords:** critically ill patients, porins, combination treatment, cross-resistance

## Abstract

Meropenem/Vaborbactam (MEM-VAB) is a novel carbapenem- β-lactamase inhibitor active against KPC-producing Enterobacteria. Herein, we evaluate the incidence of meropenem/vaborbactam-resistance among KPC-producing *K. pneumoniae* (KPC-Kp) bloodstream infection in a large Italian hospital. Meropenem/vaborbactam-resistance was found in 8% (*n* = 5) KPC-Kp, while 5% (*n* = 3) strains exhibited cross-resistance to ceftazidime/avibactam (CAZ-AVI). Genomic analysis revealed that meropenem/vaborbactam-resistance was associated with truncated OmpK35 and insertion of glycine and aspartic acid within OmpK36 at position 134–135 (GD134–135). Notably, no specific mutation was associated to cross-resistance. No specific antimicrobial treatment was related to favorable clinical outcomes, while cross-resistance was not associated to higher clinical and/or microbiological failures. Our study indicated that resistance to meropenem/vaborbactam was due to porins mutations and is associated with reduced susceptibility to both ceftazidime/avibactam and carbapenems.

## 1. Introduction

Management and treatment of patients with infections due to *Klebsiella pneumoniae* carbapenemase (KPC)-producing *K.* *pneumoniae* (KPC-Kp) is a daily challenge in clinical practice. The KPC carbapenemase is often carried out in microorganisms co-harboring different antimicrobial resistance determinants, thus conferring multiple multidrug resistant phenotypes. As a result, most antimicrobial molecules exhibit poor in vitro activity against KPC-Kp, thus reducing treatments available [1]. Combination therapies, including high-dose meropenem, colistin, fosfomycin, tigecycline, and aminoglycosides, are widely used, with suboptimal results [2,3,4].

During the past decade, significant advances were made in the development of novel β-Lactams (BL)-β-lactamase inhibitor (BLI) combinations against KPC-producing microorganisms. However, a significant challenge resides in the selection of an appropriate BL partner, as well as the development of resistance linked to the BL partner. In addition, dosing regimens for these BL-BLI combinations need to be critically evaluated [5,6,7].

Recently, antimicrobial therapy with newer agents such as ceftazidime-avibactam, cefiderocol and meropenem-vaborbactam offer promising alternatives to existing agents for the treatment of severe infections due to KPC-Kp. Vaborbactam (formerly RPX7009), is a non-β-lactam, cyclic, boronic acid-based β-lactamase inhibitor. While it is not active against metallo-β-lactamase enzymes, vaborbactam potentiates the in vitro activity of meropenem against KPC-, ESBL-, and AmpC-producing isolates [8,9]. The boron atom in vaborbactam acts as an electrophile and forms a reversible covalent bond with the catalytic serine of specific β-lactamases [10]. A previous study showed that the inhibitor vaborbactam crosses the outer membrane in *K. pneumoniae* using OmpK35 and OmpK36 porins [11].

On 20 November 2018 European Medicines Agency approved meropenem/vaborbactam (MEM/VAB) for clinical use in adult patients with difficult to treat carbapenem-resistant *Enterobacterales* (CRE) infections, satisfying an important medical issue linked to antibiotic-resistant *Enterobacterales* (https://www.ema.europa.eu/en/medicines/human/EPAR/vaborem; accessed on 31 March 2021).

MEM/VAB represents a novel antibiotic combination therapy, approved for clinical use in Europe, and indicated for complicated urinary tract infection, complicated intra-abdominal infections, hospital-acquired pneumonia, ventilator-acquired pneumonia and bacteremia associated with these infections [12].

In vitro studies demonstrated excellent activity of MEM/VAB against KPC-producers with a low ability to acquire resistance [13]. A recent clinical study showed that MEM/VAB had a superior rate of clinical cure against CRE infections in patients with bacteremia, compared to other available therapies [12]. However, limited information is still available on the efficacy of MEM/VAB in clinical practice and the in vivo evolution of emerging resistant strains [13]. Recently, in vitro studies demonstrated that mutations causing loss-of-function of OmpK35 and OmpK36 porins or reduced production could generate resistance to MEM/VAB and/or ceftazidime/avibactam (CAZ/AVI) [14,15].

The aim of this study was to investigate the incidence of meropenem/vaborbactam resistance among KPC-Kp strains isolated from patients with bloodstream infection (BSI) and characterize genomically MEM/VAB-resistant strains.

## 2. Results

We evaluated in vitro activity of MEM/VAB against 62 KPC-Kp bloodstream isolates collected from hospitalized patients admitted to our institution (S. Orsola-Malpighi Hospital, Bologna) during 2018. The retrospective clinical data revealed that 45% (28/62) of KPC-Kp bacteremic patients were hospitalized in intensive care units. At the same time, 34% (21/62) of KPC-Kp bloodstream isolates were from medicine, 10% (6/62) were in haemato-oncology, 7% (4/62) were in surgical and just 5% (3/62) were in transplantation wards. According to EUCAST breakpoints, 54 out of 62 strains (87%) were susceptible to meropenem/vaborbactam with a median MIC of 0.5 mg/L (interquartile range [IQR] 0.25–1). On the contrary, eight KPC-Kp were resistant to meropenem/vaborbactam by exhibiting a median MIC of 256 mg/L (IQR 64–256), as shown in Table 1. Notably, three of eight (37.5%) meropenem/vaborbactam-resistant KPC-Kp strains were also resistant to ceftazidime/avibactam (median MIC 32, IQR 32–256), while others meropenem/vaborbactam-resistant strains showed a CAZ/AVI MIC equal to 8 mg/L (Table 1).

Genomic analysis showed that meropenem/vaborbactam-resistant strains belonged to the internationally spreadClonal Complex 258 (ST1519, ST258 and ST512) [16] and shared similar genetic resistance determinants responsible for resistance to ß-lactams, aminoglycoside, fluoroquinolone and fosfomycin (Table 1).

In particular, the analysis of β-lactam resistance genes showed that all isolates shared the wild-type *bla_KPC-3_* gene, while five out of eight carried a *bla_SHV-182_* variant. In addition, genetic analysis showed that all three KPC-Kp strains carried *bla_KPC-3_* gene harbored in a Tn4401 isoform a.

Genetic analysis of porin genes showed that all meropenem/vaborbactam-resistant KPC-Kp strains had truncated OmpK35 due to frameshift (*n* = 8) on the porin gene and the insertion of glycine and aspartic acid within OmpK36 at position 134–135 (GD134–135).

**Figure 1:**
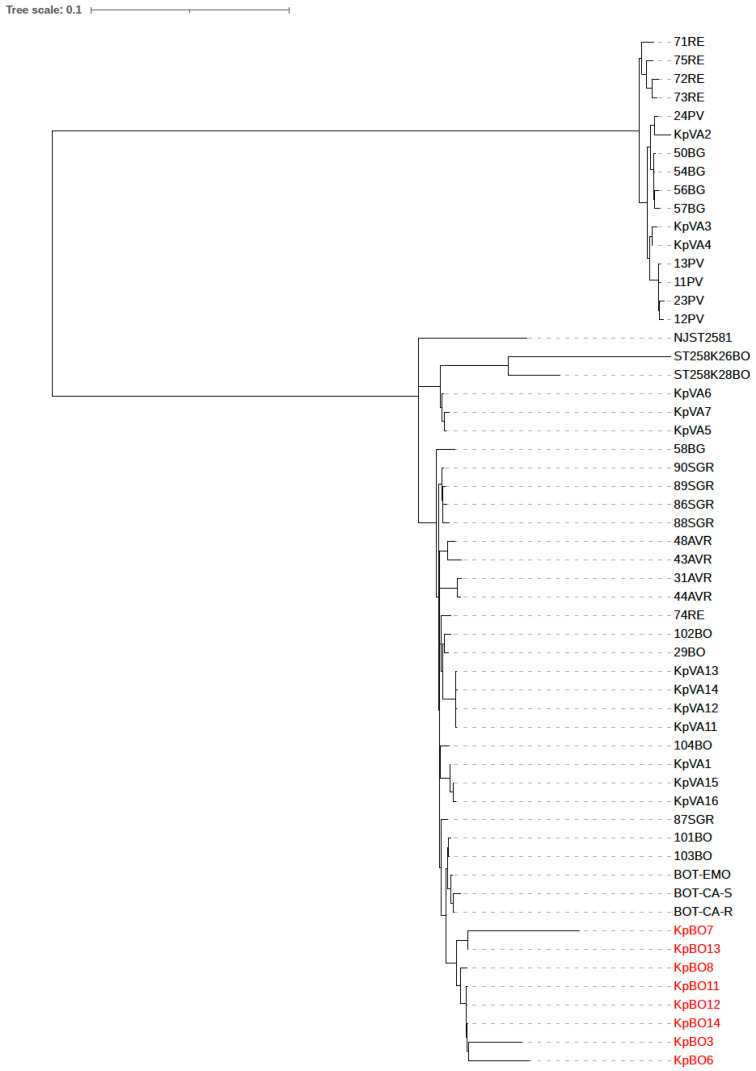
Phylogenetic tree based based on the core genome SNP of KPC-producing *K. pneumoniae* (KPC-Kp) isolated in Italy and strains included in this study. The KPC-Kp clinical isolates included in this study are highlighted in red. The genome of strain NJST2581 was used as a reference genome.

Based on genome alignment, a total of 234 SNPs in KpBO3, 336 in KpBO6, 137 in KpBO7, 137 in KpBO8, 115 in KpBO11, 111 in KpBO12, 156 in KpBO13 and 110 in KpBO14 were observed against genome of the KpBO3 strain as reference. Comparative analysis of single nucleotide polymorphisms (SNPs) and insertion-deletions (Indels) between MEM/VAB-resistant KPC-Kp strains showed that most SNPs were located intergenic regions or in hypothetical proteins and that no significant difference between MEM/VAB-resistant strains and cross-resistant (i.e., MEM/VAB and CAZ/AVI–resistant) KPC-Kp strains were observed in genes encoding antimicrobial resistance. In addition, phylogenetic analysis based on cgMLST and core-genome SNPs showed that genomes of KPC-Kp strains resistant to meropenem/vaborbactam clustered into a single clade, thus demonstrating a close relationship (Figure 1).

### Clinical Data Analysis

Retrospective clinical analysis showed that patients with BSI due to KPC-Kp resistant both to ceftazidime/avibactam and meropenem/vaborbactam had a short time of CPE colonization (median 4 days, IQR 0–7).

Clinical characteristics of patients with meropenem/vaborbactam-resistant KPC-Kp are shown in Table 2. The 30-day mortality rates were 100% (3/3) for patients with SOFA score between 10–20%, 50% (1/2) for patients with score ranging from 5% to 10% and 0% (0/3) for patients below 4.

Clinical success was achieved in 50% (4/8) of patients. None of the patients were treated with meropenem/vaborbactam-based treatment. Notably, clinical failure occurred for different antimicrobial treatments and in 33% (1/3) of meropenem/vaborbactam and ceftazidime/avibactam-resistant KPC-Kp strains. In particular, no relapsing infection was observed for all antimicrobial combination treatment. At the same time, microbiological failure was achieved in one patient treated with colistin-meropenem therapy due to persistent bacteremia.

## 3. Discussion

In this study, we evaluated the activities of meropenem/vaborbactam against 62 KPC-Kp clinical strains collected from bacteremic patients during 2018. Our findings showed that resistance to meropenem/vaborbactam emerged in 13% of KPC-Kp strains isolated from bloodstream infection. Notably, meropenem/vaborbactam resistance was associated to a high level of MICs for ceftazidime/avibactam. In particular, cross-resistance to ceftazidime/avibactam and meropenem/vaborbactam was observed in three KPC-Kp strains, which exhibited high levels of MICs for both ceftazidime/avibactam, meropenem/vaborbactam and carbapenems. 

A recent study showed that the selection of KPC-Kp mutants with reduced susceptibility to meropenem-vaborbactam was due to OmpK35 and OmpK36 porin mutations associated with an increase in the *bla_KPC_* gene copy number, whereas it was not associated with mutations in the coding region of *bla_KPC_* gene [17,18]. Our results are in agreement with these findings as we found that meropenem/vaborbactam-resistance was due to OmpK35 and OmpK36 porins depletion in KPC-Kp strains and no specific mutations were observed in the *bla_KPC_* gene [19]. At the same time, our results showed that no specific mutations were observed in KPC-Kp isolates resistant to both meropenem/vaborbactam and ceftazidime/avibactam in comparison to strains resistant to meropenem/vaborbactam alone. These results are in accordance with previous findings that showed as cross-resistance to meropenem/vaborbactam and ceftazidime/avibactam was due to loss-of-function mutations in the OmpK35 and OmpK36 porins, which are also associated to elevated MICs for β-lactams [14,15].

We observed that patients with BSI due to KPC-Kp resistant to both meropenem/vaborbactam and ceftazidime/avibactam had a short colonization time, thus suggesting that cross-resistance did not result from selection pressure due to recently antimicrobial therapy. Therefore, considering the high incidence of KPC-Kp strains in our country, it is plausible that patients acquired cross-resistant strains during hospitalization or that meropenem/vaborbactam and ceftazidime/avibactam resistance emerged after prolonged antimicrobial treatments in colonized patients [20]. Overall, clinical and microbiological failure was observed in two out of three patients with pneumonia, while no specific treatment was associated to clinical success. At the same time, microbiological failure was only observed in a colistin-meropenem combination treatment. Notably, cross-resistance to meropenem/vaborbactam and ceftazidime/avibactam was not associated with clinical or microbiological failure. 

This study is limited to a small number of patients with BSI due to meropenem/vaborbactam-resistant strains treated with different combination treatments. Further study is mandatory to evaluate the clinical outcome of different antimicrobial therapies against BSI due meropenem/vaborbactam and/or ceftazidime/avibactam–resistant strains.

In conclusion, we described the incidence of meropenem/vaborbactam resistance among KPC-Kp strains isolated from bacteremic patients. Further studies are planned to characterize specific-mutations emerging in vivo under different antimicrobial therapy and to evaluate the in vitro activity of novel antimicrobials (i.e., cefiderocol, imipenem/cilastatin/relebactam) against meropenem/vaborbactam and/or ceftazidime/avibactam-resistant strains.

## 4. Materials and Methods

### 4.1. Study Participants

Between 1 January to 31 December 2018, we collected all KPC-producing *K. pneumoniae* isolated from patients with BSIs hospitalized in the large tertiary-care university hospital in Bologna. Policlinico di Sant’Orsola (PSO) is a 1420-bed University Hospital with an average of 72,000 admissions per year. The study was conducted in accordance with the Declaration of Helsinki and its later amendments. All the samples were kept anonymous throughout the duration of the study. All consecutive adults (aged ≥18 years) with BSI due to KPC-producing *K.pnuemoniae* were included in the study. Patients were only included at the first episode of BSI due to KPC-Kp.

### 4.2. Microbiological Analysis

The blood samples were processed following the routine workflow of the microbiology laboratory of Sant’Orsola-Malpighi University Hospital. Briefly, samples were inoculated in liquid medium bottles and incubated for five days in a Bactec FX blood culture system (Becton Dickinson). The positive bottles were seeded on horse blood agar and CHROMagar Orientation (Meus, Paris, France). Moreover, positive samples were inoculated onto chocolate agar plates, which were incubated at 35–37 °C for 3 h. Species identification was performed by MALDI-TOF mass spectrometry analysis from microbial growth using Microflex instrument and MALDI Biotyper software (Bruker Daltonik, Bremen, Germany) [21]. Antimicrobial susceptibility tests were performed using an automated system MicroScan Walkaway-96 (Beckman Coulter, Brea, CA, USA). The Minimal inhibitory concentration (MIC) for meropenem/vaborbactam and ceftazidime/avibactam were tested by MIC test strip (Liofilchem, Italy). In addition, MIC value for colistin was determined by reference broth microdilution method [14]. MIC values were interpreted following European Committee for Antimicrobial Susceptibility Testing (EUCAST) breakpoints v11.0 (http://www.eucast.org/clinical_breakpoints/; accessed on 31 March 2021).

Carbapenem-resistant Enterobacteriaceae were screened for carbapenemase production following routine workflow established at the Microbiology Unit of S. Orsola-Malpighi Hospital, as previously described [22]. Briefly, carbapenemase type was determined by MALDI-TOF for 11,109 *m/z* specific peak detection for KPC [23] and/or by multiplex immunochromatographic (IC) assay NG Test CARBA 5 (NG Biotech, France) for other carbapenemase enzymes (IMP, VIM, NDM, KPC, OXA-48). In the case of discordant results between MALDI-TOF specific-peak and IC assays, and for ceftazidime/avibactam or meropnem/vaborbactam reistance phenotypes, a molecular assay (Xpert Carba-R, Cepheid) was performed to identify carbapenemase gene and to exclude Metallo-Beta-Lactamase production.

### 4.3. Whole Genome Sequencing Analysis

Whole-genome sequencing of KPC-producing *K. pneumoniae* (KPC-Kp) strains was performed to identify the molecular mechanism at the basis of meropenem/vaborbactam-resistance as previous described [24]. Briefly, libraries were prepared by the Nextera DNA flex sample preparation kit and sequenced using the Illumina Iseq100 platform (Illumina, San Diego, CA, USA) with a 2 × 150 paired end run. All read sets were evaluated by FastQC software and then assembled with SPAdes v.3.10 with careful settings. Plasmid incompatibility type and phage regions were assessed using PlasmidFinder (https://cge.cbs.dtu.dk/services/PlasmidFinder/; accessed on 31 March 2021) and PHAGE (http://phast.wishartlab.com; accessed on 31 March 2021) web tools on assembled genomes. The known antimicrobial resistance was determined by mapping the assembled contigs on the CGE server https://cge.cbs.dtu.dk/services/ (accessed on 31 March 2021).

Bacteria genomes were automatically annotated on the RAST server and Sequence type (ST) was determine by using database (BIGSdb) (http://bigsdb.web.pasteur.fr; accessed on 31 March 2021). Porin genes and Tn*4401* isoform were manually investigated by BLAST analysis. A core genome single nucleotide polymorphism (SNP) phylogeny was generated using core genome SNPs analysis using the ParSNP software [25] using draft genomes of KPC-producing *K. pneumoniae* CC258 strains isolated in Italy and complete genome of strain NJST258_1 (Accession no. NZ_CP006923.1) as reference, as previously described [25]. ParSNP was performed using settings “–c”, “-a 13” and “-x” for include all genomes, higher resolution mapping and exclusion of SNPs located in regions of recombination. The maximum likelihood tree was constructed from final alignment of core-genome SNPs using FastTree with generalized time-reversible mode and visualized by using iTOL software [26]. Additionally, epidemioligcal analysis was confirmed by using core genome MLST analysis [27]. Single nucleotide polymorphisms (SNPs) and insertion-deletions (Indels) between MEM/VAB-resistant KPC-Kp genomes were investigated using Breseq [28].

### 4.4. Data Availability

The sequencing reads generated during the current study are available via the NIH Sequence Read Archive (SRA) via Bioproject PRJNA722151.

### 4.5. Clinical Data and Statistical Analysis

The severity of illness during the onset of BSI was calculated by Sequential Organ Failure Assessment score (SOFA score), as previously described [29,30].

The clinical outcome was defined as all-cause mortality at day 30 after BSI onset (index BC collection day). The microbiologic failure was defined as isolation of KPC-Kp strain with similar antimicrobial susceptibility pattern following 30 days of target antimicrobial treatment. The relapse infection was defined as growth of the same organism with a similar antimicrobial resistance pattern to KPC-producing *K. Pneumoniae* isolated in the first BSI after the end of therapy, but before day 30.

The results (continuous variables non normally distributed) were analyzed as the median and interquartile range (IQR) or as percentages of the group from which they were derived (categorical variables). 

## Figures and Tables

**Table 1:** Phenotypic and genotypic characteristics of meropenem/vaborbactam-resistant KPC-producing *Klebsiella pneumoniae* strains.

Isolate	Patient	MIC (mg/L)				ST	Genetic Determinants				*Porins*		Plasmid_Replicons (InC)
		MEM	CAZ/AVI	MEM/VAB	CST		Beta-lactams	Aminoglycoside	Fluoroquinolone	Sulfonamide	*OmpK35*	*OmpK36*	
KpBO3	1	256	32	256	0.25	512	*bla_KPC-3_, bla_SHV-11_*	*aac(6’)-Ib*	*oqxA, oqxB, aac(6’)Ib-cr*	*sul1*	truncated	GD134–135	IncFIB (K), IncFIB(pKPHS1), IncX3, ColRNAI
KpBO6	2	256	16	256	0.5	258	*bla_KPC-3_, bla_SHV-12_*	*aadA2, aph(3’)-Ia, aac(6’)Ib-cr*	*oqxA, oqxB, aac(6’)Ib-cr*	*sul1*	truncated	GD134–135	IncFIIK, IncFIB(K), IncX3, ColRNAI
KpBO7	3	256	≥256	256	0.5	1519	*bla_KPC-3_, bla_TEM-1A,_ bla_OXA-9_, bla_SHV-11_*	*aadA2, aph(3’)-Ia, aac(6’)Ib-cr*	*oqxA, oqxB, aac(6’)Ib-cr*	*sul1*	truncated	GD134–135	IncFIB (pQIL), IncFIB (pKPSH1), IncFIB(K), IncFII(K), IncX3, ColRNAI, Col(BS512)
KpBO8	4	32	8	48	0.5	512	*bla*_KPC-3_ *, bla_SHV182_*_,_ *bla_TEM-1A,_ bla_OXA-9_*	*aadA2, aph(3’)-Ia, aac(6’)-Ib*	*oqxA, oqxB, aac(6’)Ib-cr*	*sul1*	truncated	GD134–135	IncFIB (pQIL), IncFIB (pKPSH1), IncFIB(K), IncFII(K), ColRNAI
KpBO11	5	256	8	256	0.25	512	*bla_KPC-3_, bla_SHV182_*	*aadA2, aph(3’)-Ia, aac(6’)-Ib*	*oqxA, oqxB, aac(6’)Ib-cr*	*sul1*	truncated	GD134–135	IncFIB (pKPSH1), IncFIB(K), IncFII(K), ColRNAI, IncX3
KpBO12	6	256	8	256	0.5	512	*bla_KPC-3_, bla_SHV182_*	*aadA2, aph(3’)-Ia, aac(6’)-Ib*	*oqxB, oqxA, aac(6’)Ib-cr*	*sul1*	truncated	GD134–135	IncFIB (pKPSH1), IncFIB(K), IncFII(K), ColRNAI, IncX3
KpBO13	7	32	8	256	0.5	1519	*bla_KPC-3_, bla_SHV182,_ bla_OXA-9_*	*aadA2, aac(6’)-Ib*	*oqxB, oqxA, aac(6’)Ib-cr*	*sul1*	truncated	GD134–135	IncFIB (pQIL), IncFIB (pKPSH1), IncFIB(K), IncFII(K), ColRNAI, IncX3, Col(BS512)
KpBO14	8	256	8	256	0.25	512	*bla_KPC-3_, bla_SHV182_*	*aac(6’)-Ib*	*oqxA, oqxB, aac(6’)Ib-cr*	-	truncated	GD134–135	IncFIB (pKPSH1), IncFIB(K), ColRNAI, IncX3

Abbreviations: MEM, meropenem; CAZ/AVI, ceftazidime/avibactam; MEM/VAB, meropenem/vaborbactam; CST, colistin; ST, sequence type.

**Table 2:** Clinical characteristics of patients with bloodstream infection due to Meropenem/Vaborbactam-resistant KPC-producing *Klebsiella pneumoniae* (KPC-Kp) strains.

Patient	Isolate	Colonization Days’ Prior Infection	SOFA	Initial Infection	Previous Treatment (Days)	Time of Isolation after Initial Treatment (Days)	Antimicrobial Combination Therapy (Days)	Risk Factors	Clinical Outcome at 30 Days	Microbiological Outcome at 30 Days (Days)	Relapse Infection
1	KpBO3	0	13	Abdominal infection	Ceftazidime/Avibactam (12), Meropenem (32)	0	Meropenem-Colistin-Tigecycline (3)	-	Failure	NA	NA
2	KpBO6	4	2	Pneumonia	Meropenem-Colistin (27), Meropenem-Tigecycline (19)	0	Meropenem-Colistin (24)	-	Success	Success	None
3	KpBO7	7	1	CVC-related	Amoxicillin/Clavulanic acid (11)	1	Meropenem-Colistin (13)	CVVH	Success	Success	None
4	KpBO8	60	6	CVC-related	Oxacillin (11), Ertapenem (13)	0	Meropenem-Ceftazidime/Avibactam (24)	CKD	Success	Success	None
5	KpBO11	18	12	Pneumonia	Oxacillin (8), Piperacillin/Tazobactam (34)	15	Meropenem-Colistin (18)	-	Failure	Failure (15)	NA
6	KpBO12	3	20	Biliary infection	Piperacillin/Tazobactam (3), Meropenem-Tigecycline (3)	10	Meropenem-Tigecycline (1)	CKD, CVVH	Failure	NA	NA
7	KpBO13	5	5	Pneumonia	Piperacillin/Tazobactam (16), Meropenem-Tigecycline (29)	1	Meropenem-Ceftazidime/Avibactam (14)	CKD	Failure	NA	NA
8	KpBO14	60	3	Urinary infection	Amoxicillin/Clavulanic acid (20), Meropenem (19)	0	Meropenem-Ceftazidime/Avibactam (14)	CKD	Success	Success	None

Abbreviations: Bloodstream infection, BSI; not changed, NC; Central Venous Catheter, CVC; Continuous Venous Hemofiltration, CVVH; Chronic Kidney Disease, CKD; not applicable, NA.

## Data Availability

The sequencing reads are available at the NIH Sequence Read Archive (SRA) via Bioproject PRJNA722151.
